# Toward a Better Understanding of Quality Social Connections. Comment on “Quality Social Connection as an Active Ingredient in Digital Interventions for Young People With Depression and Anxiety: Systematic Scoping Review and Meta-analysis”

**DOI:** 10.2196/36739

**Published:** 2022-03-11

**Authors:** Huachu Deng, Xingan Qin

**Affiliations:** 1 Department of Gastrointestinal and Gland Surgery First Affiliated Hospital of Guangxi Medical University Nanning China

**Keywords:** mental health, digital interventions, young people, quality social connection, depression, anxiety, systematic review, meta-analysis, patient and public involvement, mobile phone

We read the paper by Dewa et al [1] with interest. The authors performed a meta-analysis to conceptualize, appraise, and synthesize evidence on quality social connection (QSC) within digital interventions (D-QSC) and the impact on depression and anxiety outcomes for young people aged 14-24 years. They demonstrated that “D-QSC is an important and underconsidered component for youth depression and anxiety outcomes. Researchers and developers should consider targeting improved QSC between clinicians and young people within digital interventions for depression. Future research should build on our framework to further examine relationships among individual attributes of QSC, various digital interventions, and different populations.” After carefully reading, I wish to put forth the following suggestions.

Two studies [2,3] in Table 2 (“Data extraction and quality assessment of included studies”) were created by the same author team (Radovic et al) with similar characteristics (year, country, study design, setting and participants, digital intervention, and outcomes and measures). This duplicate inclusion of data would affect the credibility of the final results of the meta-analysis. I recommend that the authors exclude duplicate works in the meta-analysis and that a correction and reliable checking of the data insertion is required.

Due to these mistakes, I suggest that the authors further refine the inclusion criteria for the included studies to avoid duplication of inclusion.

## Abbreviations

D-QSC: quality social connection within digital interventions
QSC: quality social connection
